# Genome characteristics and the ODV proteome of a second distinct alphabaculovirus from *Spodoptera litura*

**DOI:** 10.1186/s12864-024-09989-3

**Published:** 2024-01-22

**Authors:** Weisong Gao, Xingjian Liu, Xintao Gao, Tong Wu, Shuang Wei, Zhifang Zhang, Huan Zhang, Yinü Li

**Affiliations:** 1 National Key Laboratory of Agricultural Microbiology, Biotechnology Research Institute, Chinese Academy of Agricultural Sciences, 100081 Beijing, China; 2 State Key Laboratory of Integrated Management of Pest Insects and Rodents, Institute of Zoology, Chinese Academy of Sciences, 100101 Beijing, China

**Keywords:** *Spodoptera litura* Nucleopolyhedrovirus, Baculovirus, Genome sequence, Virus species demarcation criteria, ODV proteome

## Abstract

**Background:**

*Spodoptera litura* is a harmful pest that feeds on more than 80 species of plants, and can be infected and killed by *Spodoptera litura* nucleopolyhedrovirus (SpltNPV). SpltNPV-C3 is a type C SpltNPV clone, that was observed and collected in Japan. Compared with type A or type B SpltNPVs, SpltNPV-C3 can cause the rapid mortality of *S. litura* larvae.

**Methods:**

In this study, occlusion bodies (OBs) and occlusion-derived viruses (ODVs) of SpltNPV-C3 were purified, and OBs were observed by scanning electron microscopy (SEM). ODVs were observed under a transmission electron microscope (TEM).

**Results:**

Both OBs and ODVs exhibit morphological characteristics typical of nucleopolyhedroviruses (NPVs).The genome of SpltNPV-C3 was sequenced and analyzed; the total length was 148,634 bp (GenBank accession 780,426,which was submitted as SpltNPV-II), with a G + C content of 45%. A total of 149 predicted ORFs were found. A phylogenetic tree of 90 baculoviruses was constructed based on core baculovirus genes. LC‒MS/MS was used to analyze the proteins of SpltNPV-C3; 34 proteins were found in the purified ODVs, 15 of which were core proteins. The structure of the complexes formed by *per os* infectivity factors 1, 2, 3 and 4 (PIF-1, PIF-2, PIF-3 and PIF-4) was predicted with the help of the AlphaFold multimer tool and predicted conserved sequences in PIF-3. SpltNPV-C3 is a valuable species because of its virulence, and the analysis of its genome and proteins in this research will be beneficial for pest control efforts.

**Supplementary Information:**

The online version contains supplementary material available at 10.1186/s12864-024-09989-3.

## Background

Baculoviridae is the largest viral family; it consists of rod-shaped viruses specific to arthropods. It is a type of enveloped virus with a circular double-stranded DNA genome ranging in size from 80 to 180 kb [1] and containing 100 to 180 open reading frames [2]. Baculoviruses were among the first species of insect viruses discovered, and more than 600 insect species across 7 orders, such as Lepidoptera, Hymenoptera, and Diptera, have been reported to be infected by baculoviruses. To date, 91 complete genomes have been recorded in National Center for Biotechnology Information (NCBI) database; these include four genera: *Alphabaculovirus* (61), *Betabaculovirus* (26), *Gammabaculovirus* (3), and *Deltabaculovirus* (1) [3].

Over the course of coevolution, baculoviruses have evolved two types of virions, budded viruses (BVs) and occlusion-derived viruses (ODVs), during their life cycle to enhance the ability to infect the host. One or more ODVs are released from occlusion bodies (OBs) in an alkaline environment such as the gut of an insect; ODVs are thus released, and subsequently infect midgut epithelial cells [4]. After infecting midgut epithelial cells, BVs are packaged and released to disseminate systemic infection from cell to cell. In the next several days, the larvae dissolve from the inside and release the OBs. Because of this trait, since the 1940s, baculoviruses have been studied as biopesticides in crop fields. Although baculoviruses cannot kill insect larvae quickly, baculoviruses are still targeted, environmentally friendly and low-cost biopesticide.

*Spodoptera litura*, which belongs to the family *Noctuidae* and is called the tobacco cutworm or cotton leafworm, is a nocturnal moth found across Asia, Oceania, and the Indian subcontinent. Larvae eat indiscriminately and voraciously, and thus pose a threat to cash crops. Using chemical insecticides is not friendly to the environment, and insecticidal lamps are mainly aimed at imagines. In contrast, using baculoviruses as biopesticides is a good choice. *Spodoptera litura* nucleopolyhedrovirus (SpltNPV) is widely found in Central Asia, including China, Japan and Pakistan [5], and has been successfully applied as a commercial biopesticide against defoliating insects in China. An analysis of samples collected by Kamiya in Japan identified three NPV types, as type A, type B, and type C. A clone from type C SpltNPV called SpltNPV-C3 could cause more rapid mortality of *S. litura* larvae than type A or type B SpltNPV [6].

It is important to analyze the genome and predict the structure of proteins information for determining the lethality of baculovirus and identifying the host domain. In this study, the genome of SpltNPV-C3 was analyzed, OBs and ODVs were purified and observed via electron microscopy, proteins were separated via LC‒MS/MS, and simulated structures were connected and associated with oral infection.

## Materials and methods

### Virus preparation and purification

SpltNPV-C3was a gift from Jiang Zhu (Soochow University), and was originally obtained from Katsumi Kamiya (Gifu Prefectural Institute for Bio-Industrial Technology, Minokamo, Japan).

*S. litura* larval corpses were collected, ground in a mortar with PBS and filtered through cheesecloth. The collected filtrate was centrifuged at 500 rpm (30 × g) for 10 min (Hitachi CF15RX II), followed by pelleting with centrifugation via 8000 rpm (7100 × g) for 30 min, washing the sediment at 3000 rpm (1000 × g) for 20 min with PBS three times, collecting the sediment at 8000 rpm for 30 min, and storing it at 4 ℃.

ODVs were collected from liquefied larvae. Freshly purified OBs of SpltNPV-C3 suspended in ddH_2_O were incubated with an equal volume of lysis buffer (0.3 M Na_2_CO_3_, 0.5 M NaCl, and 0.03 M EDTA, pH 9.5) at 37 °C for 10 min. The pH was adjusted to 7.5 with 0.1 M HCl. The viral OBs were purified by differential centrifugation. The released ODVs were purified via a 30–60% discontinuous sucrose gradient by centrifugation at 100,000 × g (Hitachi CS150GX II) for 90 min at 4 ℃. The collected ODVs were washed in 0.1× TE (10 mM Tris–HCl and 1 mM EDTA, pH 7.5) by centrifugation at 40,000 × g at 4 ℃ for 1 h. The sediment was resuspended in 0.1× TE [7]. 

### Electron microscopy observation

OBs of SpltNPV-C3 were observed by scanning electron microscopy (SEM; Hitachi SU8010), and ODVs of SpltNPV-C3 were observed by transmission electron microscopy (TEM; Hitachi HT7700) according to standard methods [8].

### DNA sequencing and analysis

A random genomic library of SpltNPV-C3 was constructed according to the partial filling-in method (Chen et al., 2009) [9]. ORFs were defined using ORF Finder (http://www.ncbi.nlm.nih.gov/gorf/gorf.html). DNA and protein comparisons were performed using BLAST (http://blast.ncbi.nlm.nih.gov/Blast.cgi). Protein homology and translated ORFs were identified by the HHpred webserver [10, 11]. Multiple alignments and percentage identities were obtained using ClustalW. Putative ORFs were screened as described previously [12]. Phylogenetic analysis of SpltNPV-C3 was conducted through a phylogenetic tree based on the amino acid sequences of the core genes of *Baculoviridae* available in the ICTV (https://talk.ictvonline.org/ictv-reports/ictv_online_report/dsdna-viruses/w/baculoviridae) using the maximum likelihood method and tested by the bootstrap method in MEGA X. *Late expression factor 8* (*lef-8*), *late expression factor 9* (*lef-9*) and *polyhedrin* (*polh*) were seriated and used to calculate the genetic distances via MEGA (Kimura two-parameter model) [13].

### Protein separation and in-gel digestion

The proteins of the SpltNPV-C3 OBs were separated via SDS‒PAGE using an 8–15% gradient gel. The protein bands were collected into a 1.5 mL centrifuge tube for LC‒MS/MS analysis (Thermo Fisher Scientific, MA, USA). LC‒MS/MS analysis and protein identification were performed by Shanghai Omicsolution Co. The raw files of the MS spectra were searched against the putative protein database SpltNPV-C3 (NC_011616).

### Protein structure simulation

The amino acid sequences used were found in the NCBI genome database Complete genomes: Baculoviridae (nih.gov). The 3D structure was simulated by the AlphaFold Multimer tool. Conserved sequences were estimated by The ConSurf Server (tau.ac.il).

## Results and discussion

### Electron microscopy observation

**Fig. 1:**
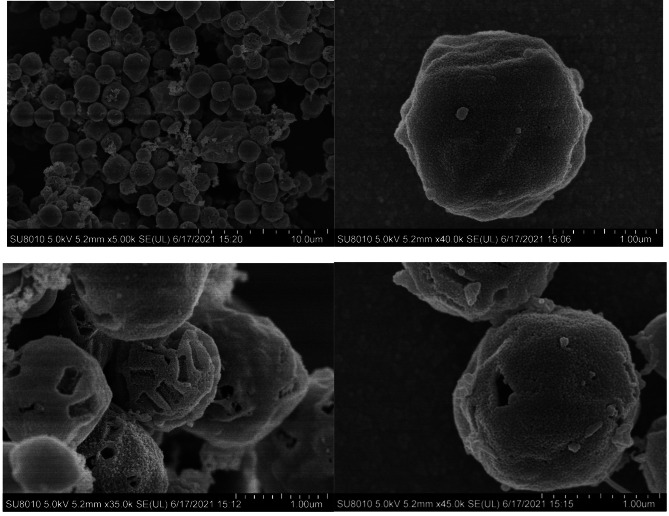
Scanning electron micrographs of SpltNPV-C3 OBs. The magnification is indicated at the bottom of the image. **A:** 5 000×, **B:** 40 000×, **C:** 35 000×, **D:** 45 000×

**Fig. 2:**
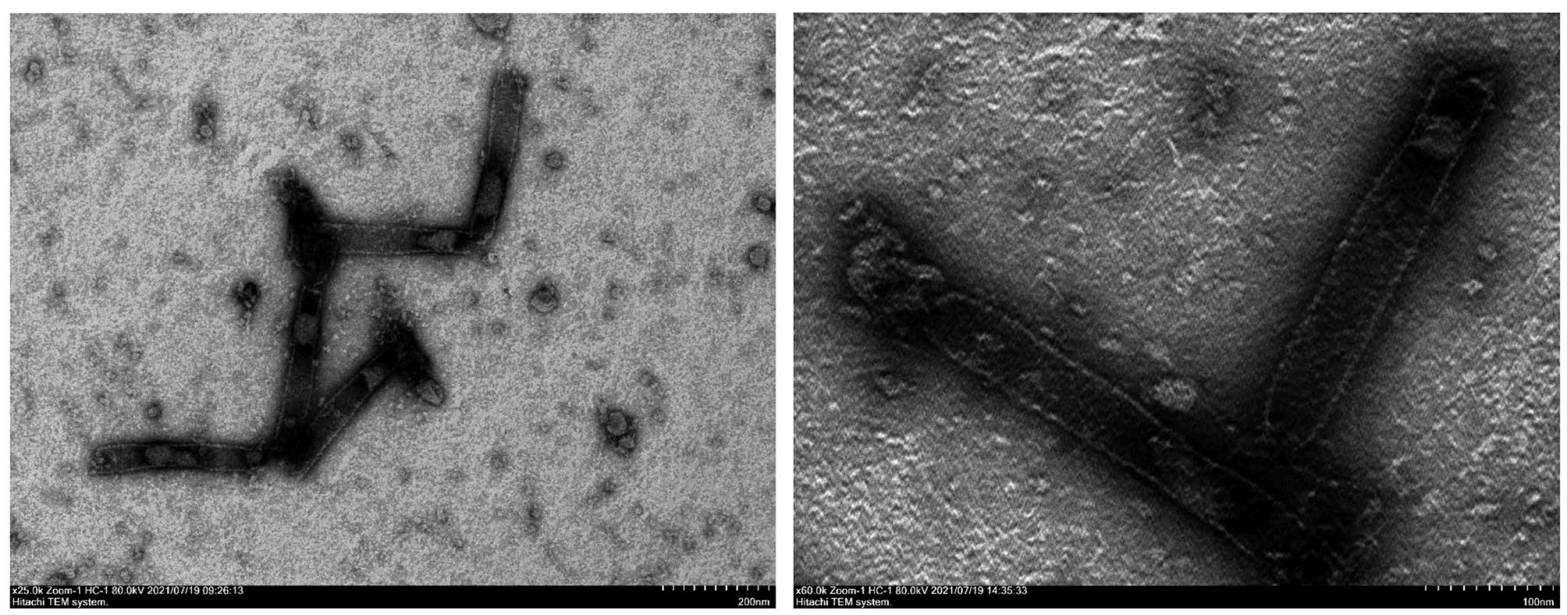
Transmission electron micrographs of SpltNPV-C3 ODVs. Magnification is indicated at the bottom of the image. **A:** 60 000×, **B:** 25 000×. The images were created using electron micrographs, and the whole appearance of SpltNPV-C3 OBs is clearly shown. Most OBs consist of polyhedrin, which is important for protecting baculoviruses from harsh environments until the next host is found. Baculoviruses can be used as delivery vectors since their genome can contain a long exogenous gene, and viruses produced from larvae can survive against complement attack [14], whereas those packaged by cells cannot survive [15]. There is an obvious difference between these two production methods; viruses in larvae experience the whole cycle of baculovirus infection and produce OBs when they exit the larval body

Polyhedrin envelops ODVs to protect them from extraneous harmful environmental risks. Previous research has shown the physical form of baculoviruses. With the help of SEM, our results showed that the OBs of SpltNPV-C3 are packaged with spherical polyhedra that have an uneven surface and are approximately 1.5 μm in diameter, in accordance with the standard mode of *Alphabaculovirus*. The structure of the virus is shown in Figs. 1 and 2.

### Sequence and genome characteristics of SpltNPV-C3

The whole genome of SpltNPV-C3 is 148,634 bp (GenBank accession 780,426, which was submitted as SpltNPV-II) with a G + C content of 45%. The SpltNPV-C3 genome is 9 kb longer than the SpltNPV-G2 genome (139,342 bp) [16] and 4 kb longer than that of the first sequenced baculovirus, AcMNPV (*Autographa California* multiple nucleopolyhedroviruses) (133,894 bp). According to the general criteria for discriminating ORFs [17, 18], 149 ORFs were found. The number of predicted ORFs and the length of the genome are similar to those of AgseNPV (*Agrotis segetum* nucleopolyhedrovirus) (151). Among these predicted ORFs, 24 contain early promoter motifs (a CAG/TT motif downstream of the TATA box and within 180 bp upstream of the start codon ATG), 55 contain late promoter motifs (an (A/T/G) TAAG motif downstream within 180 bp upstream of the start codon ATG), and 18 contain early and late promoter motifs, implying that these genes can be transcribed in the early and late stages of viral infection; 51 have no typical motifs for distinguishing early or late characteristics and are difficult to classify. The reading frames and homologous repeat regions are shown in Table 1.

**Table 1:** SpltNPV-C3 predicted open reading frames (ORFs) and homologous repeat regions (*hrs*)

Predicted ORF	Name	Position	Length(aa)	Promoter	Homologous ORF#/amino acid identity(%)			
					AcMNPV	SpltNPV-G2	SeMNPV	SperNPV
1	polyhedrin	1 > 741	246	L	8/85	1/83	1/98	1/100
2	orf1629	816 < 2291	491	L	9/26	2/-	2/62	2/89
3	pk1	2293 > 3189	298		10/43	3/43	3/85	3/97
4	hoar	3231 < 5420	729	E	-	-	4/47	4/85
5	unknown	5808 > 6017	69	E	-	-	-	5/100
6	unknown	6388 > 7896	502	E	-	-	5/36	7/86
7	odv-e56	8131 > 9234	367	L	148/47	17/47	6/85	8/98
8	me53	9499 > 10,629	376		139/28	27/24	7/75	9/99
*hr*1	-	10,888–12,656	-	-	-	-	-	-
9	unknown	13,083 < 13,457	124	E	-	-	-	10/93
*hr*2	-	13,574–14,209	-	-	-	-	-	-
10	F protein	14,617 > 16,638	673	E,L	23/20	136/34	8/80	11/98
11	gp16	16,838 < 17,128	96	L	130/33	-	9/86	12/99
12	p24	17,157 < 17,846	229	L	129/37	116/43	10/78	13/99
13	unknown	17,933 > 18,265	110	L	-	-	11/72	14/98
14	lef-2	18,228 > 18,863	211		6/40	114/38	12/79	15/98
15	p38.7	18,914 < 20,017	367		13/30	128/30	13/75	16/97
16	lef-1	20,017 < 20,670	217		14/42	129/42	14/80	17/83
17	cath	20,767 < 21,780	337	L	127/53	54/52	16/88	19/90
18	chitinase	21,818 > 23,569	583	L	126/65	42/57	19/85	21/90
19	unknown	23,616 < 24,038	140		-	-	20/27	22/84
20	unknown	24,148 > 24,636	162	E	-	-	21/68	23/95
21	unknown	24,852 > 26,243	463	L	-	105/35	23/68,	24/96
22	gp37	26,334 > 27,146	270	L	64/56	32/53	25/88	25/99
23	ptp2	27,143 < 27,652	169	L	-	-	26/81	26/97
24	egt	27,757 > 29,316	519	E	15/48	121/49	27/88	27/97
25	unknown	29,443 > 29,952	169	E	-	-	28/79	28/99
26	unknown	30,127 > 30,297	56		-	-	-	-
27	unknown	30,599 > 30,838	79		-	-	-	-
28	unknown	30,965 > 31,159	64	E	-	-	28/85	-
29	unknown	31,186 > 31,821	211	L	17/	119/31	29/68	29/95
30	unknown	31,858 < 34,623	921	E	-	118/25	30/71	30/96
31	unknown	34,602 > 35,216	204		-	-	31/72	31/96
32	pkip	35,271 > 35,774	167	L	24/24	133/29	32/61	32/97
33	unknown	35,813 < 36,148	111	E			33/61	33/98
34	unknown	36,602 > 36,802	66		-	-	-	
35	arif1	36,821 < 37,126	101		-	-	34/78	34/97
36	PIF-2	36,957 > 38,219	420		22/52	135/58	35/92	35/98
37	PIF-1	38,245 > 39,861	538		119/46	124/48	36/82	36/95
38	unknown	39,818 > 40,060	80		-	-	37/81	37/99
39	fgf	40,180 < 41,439	419		32/24	122/31	38/66	38/93
40	unknown	41,366 > 41,533	55	E	-	-	39/58	39/85
41	unknown	41,692 > 42,501	269	E	-	-	40/73	40/97
42	alk exo	42,513 < 43,736	407	L	133/37	109/42	41/70	41/96
*hr*3	-	43,742–44,612	-	-	-	-	-	-
43	unknown	44,614 < 44,952	112	E	19/25	112/30	42/75	42/99
44	unknown	44,951 > 46,105	384	E,L	18/24	113/33	43/84	43/97
45	unknown	46,170 < 46,562	130		-	-	44/81	44/99
46	rr2b	46,689 > 47,630	313	E,L	-	63/57	45/93	45/100
47	Calyx/pep	47,679 < 48,680	333	L	131/35	132/44	46/91	46/100
48	unknown	48,784 < 49,110	108		117/43	-	47/68	47/95
49	unknown	49,156 < 49,500	114		-	108/28	-	48/96
50	unknown	49,622 < 50,278	218		-	-	-	49/93
51	sod	50,455 < 50,910	151	L	31/68	30/68	48/93	50/99
52	unknown	50,996 > 51,442	148	E	-	-	49/36	51/32
53	PIF-3	51,464 > 52,105	213		115/44	107/51	50/84	52/98
54	unknown	52,102 > 52,545	147		-	-	51/73	53/97
55	unknown	52,610 > 54,115	501		-	102/22	52/74	54/99
56	unknown	54,166 > 54,840	224	L	106/63,	101/50	53/88	55/98
57	unknown	54,922 < 56,019	365		33/35	100/28	54/69	56/97
*hr*4	-	56,264–58,475	-	-	-	-	-	-
58	dUTPase	58,551 > 58,982	143		-	-	55/93	57/98
59	unknown	59,092 > 59,736	214	E,L	-	-	-	58/82
60	p13	59,685 > 60,605	306	L	99/54	-	56/89	59/97
61	odv-e66	60,698 > 62,749	683	E,L	46/31	98/41	57/57	60/97
62	unknown	62,746 < 63,087	113	L	108/41	97/45	58/85	61/99
63	odv-ec43	63,100 < 64,170	356		109/44	96/47	59/98	62/100
64	PIF-7	64,154 < 64,333	59		110/38	95/48	60/88	63/100
65	p87	64,330 < 66,045	571	E	104/22	94/33	61/66	64/97
66	p48	66,114 > 67,241	375	L	103/51	93/59	62/91	65/99
67	p12	67,231 > 67,530	99	L	102/28	92/33	63/78	66/99
68	p40	67,559 > 68,707	382	E,L	101/42	91/43	64/89	67/98
69	p6.9	68,771 > 69,013	80	L	100/58	90/56	65/83	68/96
70	lef-5	69,035 < 69,862	275		99/58	89/50	66/92	69/99
71	38 K	69,761 > 70,660	299	L	98/45	88/51	67/87	70/99
72	unknown	70,684 > 71,166	160	L	-	-	68/53	-
73	*bro*	71,073 < 72,182	369		Feb-40	30/31,	13/23	72/92
74	unknown	72,252 < 72,671	139		-	-	-	73/95
75	PIF-4	72,691 < 73,200	169	L	96/52	87/54	69/89	74/100
76	helicase	73,169 > 76,822	1217	L	95/41	86/42	70/85	75/99
77	odv-e25	76,926 < 77,576	216	L	94/46	85/60	71/41	76/99
78	p18	77,573 < 78,043	156		93/-	-	72/92	77/100
79	p33	78,049 > 78,807	252	E	92/53	83/51	73/96	78/99
*hr*5	-	78,945–79,333	-	-	-	-	-	-
80	lef-4	79,368 < 80,747	459		90/47	82/48	74/78	79/99
81	vp39	80,746 > 81,735	329	E,L	89/42	81/44	75/94	80/99
82	cg30	82,100 > 83,476	458	E	88/-	-	76/37	81/87
83	p95	83,695 < 86,157	820	L	83/40	79/34	77/77	82/96
84	tlp20	86,126 > 86,755	209	L	82/28	Feb-78	78/69	83/86
85	unknown	86,514 > 87,302	262	L	81/49	77/52	79/81	84/96
86	gp41	87,268 > 88,278	336	L	80/51	76/58	80/95	85/99
87	unknown	88,287 > 88,658	123	E,L	78/33	May-69	81/62	86/96
88	vlf1	88,660 > 89,781	373	E,L	77/66	74/62	82/98	87/99
89	cg30	90,501 > 90,803	100		-	-	76/25	-
90	unknown	91,116 > 91,814	232		-	-	hear52	-
91	p26	92,069 < 92,824	251	E	136/25	-	87/84	88/98
92	iap2	92,886 < 93,827	313	L	71/31	64/28	88/73	89/95
93	MTase	93,634 < 94,518	294		69/48	65/41	89/79	90/97
94	PIF-6	94,502 < 94,888	128		68/48	-	90/82	91/99
95	lef-3	94,887 > 96,062	391	L	67/28	67/28	91/64	92/94
96	desmo	96,043 < 98,277	744		66/22	68/24	92/68	93/96
97	DNA pol	98,276 > 101,440	1054	E	65/46	69/53	93/86	94/97
98	unknown	101,476 < 101,865	129	L	75/25	72/34	94/87	95/100
99	unknown	101,880 < 102,137	85	E,L	76/43	73/55	95/96	96/100
100	unknown	102,260 > 102,580	106	L	150/31	-	96/65	97/96
101	lef-9	102,667 < 104,157	496		62/63	59/69	97/95	98/98
102	fp25k	104,246 > 104,836	196	E,L	61/60	57/72	98/96	99/100
103	p94	105,059 > 107,248	729	L	134/23	-	99/66	100/51
104	chaB2	107,594 > 107,857	87	E,L	60/48	52/29	100/89	101/100
105	chaB1	107,871 > 108,455	194	L	59/52	52/37	101/60	102/93
106	unknown	108,448 < 108,993	181		57/39	51/31	102/76	103/99
107	unknown	109,143 < 109,463	106		56/-	-	103/73	104/95
108	unknown	109,360 > 109,575	71		-	-	-	-
109	unknown	109,381 < 109,584	67	L	55/38	50/40	104/91	105/98
110	vp1054	109,704 < 110,729	341	L	54/41	49/46	105/83	106/97
111	lef-10	110,590 < 110,817	75	L	53a/48	48/50	106/81	107/97
112	unknown	110,801 > 111,004	67	L	-	47/38	-	108/97
113	unknown	111,026 > 111,994	322	L	-	46/27	107/69	109/97
114	unknown	112,094 < 112,507	137	L	53/48	45/48	108/88	110/100
115	unknown	112,570 > 113,103	177		52/23	44/27	109/68	111/91
*hr*6	-	113,266–114,152	-	-	-	-	-	-
116	iap-3	114,369 > 115,241	290	L	27/30	-	110/69	113/95
117	bjdp	115,288 < 116,490	400		-	39/25	111/66	114/97
118	lef-8	116,409 > 119,180	923		50/61	38/62	112/89	115/98
119	unknown	119,313 < 119,480	55	L	43/44	-	113/78	116/98
120	odv-e66	119,515 < 121,581	688	E,L	46/26	98/29	114/77,	117/97
121	p47	121,634 > 122,851	405		40/54	36/57	115/89	118/99
122	unknown	122,942 > 123,577	211	E	-	-	116/32	119/90
123	unknown	123,649 > 124,230	193	E	-	-	117/66	120/94
124	ADPRase	124,307 > 125,053	248	E,L	38/62	35/52	118/95	121/99
125	lef-11	124,936 > 125,367	143	L	37/36	34/39	119/81	122/91
126	39 K	125,321 > 126,265	314		36/32	33/25	120/65	123/93
127	unknown	126,317 > 126,610	97		-	-	121/55	124/91
128	unknown	126,667 < 126,873	68		-	-	122/85	125/98
129	ubiquitin	126,877 < 127,113	78	L	35/-	32/91	123/94	126/99
130	unknown	127,180 > 127,776	198	L	34/38	18/55	124/79	127/96
*hr*7	-	127,816–130,509	-	-	-	-	-	-
131	unknown	130,536 < 130,931	131	L	26/35	31/39	125/73	128/96
132	dbp	131,040 > 132,008	322		25/28	30/27	126/74	129/85
133	lef-6	132,031 > 132,516	161	L	28/34	29/27	127/61	130/85
134	unknown	132,561 < 132,821	86		29/31	28/41	128/86	131/99
135	p26	133,058 > 133,861	267	L	136/35	-	129/84	132/99
136	p10	133,912 > 134,190	92	E,L	-	19/45	130/84	133/91
137	p74	134,271 < 136,220	649	E,L	138/54	21/51	131/87	134/99
138	unknown	136,326 > 136,592	88	E	-	-	-	135/99
139	unknown	136,794 > 137,213	139	E	-	-	20/32	136/100
140	*bro*	137,274 < 138,791	505		Feb-27	125/69	-	137/71
141	ie1	138,962 < 141,085	707	E	147/31	16/27	132/64	138/96
142	unknown	140,981 > 141,748	255	E,L	146/32	15/34	133/72	139/93
143	unknown	141,878 < 142,156	92	L	-	-	134/91	140/98
144	odv-ec27	142,162 < 143,004	280	L	144/55	13/50	135/96	141/99
145	odv-e18	143,088 < 143,333	81		143/51	Dec-59	136/93	142/100
146	p49	143,362 < 144,744	460	L	142/50	Nov-51	137/94	143/99
147	ie0	144,756 < 145,475	239	L	141/28	Aug-39	138/86	144/99
148	rr1	145,638 < 148,352	904	E,L	-	23/53	139/77	145/94
149	unknown	148,361 < 148,543	60	L	-	-	-	146/90

Note: Putative SpltNPV-C3 predicted ORFs are listed in Column 1, and the gene homologs are listed in Column 2. Column 3 indicates the ORF location and transcriptional direction in the SpltNPV-C3 genome. Column 4 indicates the number of amino acids. Column 5 indicates the presence of early (E) and/or late (L) promoters located upstream of the start codon of each ORF. E indicates a TATA sequence followed by a CAGT or CATT mRNA start site sequence 20–40 nucleotides downstream, 180 bp upstream of the start codon. L indicates the presence of a (A/T/G) TAAG sequence. Columns 6–9 list the homologous ORF and percent amino acid identity from AcMNPV, SpltNPV-G2, SeMNPV (*Spodoptera exigua* multiple nucleopolyhedrovirus), and SperNPV (*Spodoptera eridania* nucleopolyhedrovirus), respectively

### Comparison of SpltNPV-C3 predicted ORFs to those of other baculoviruses

By comparing the gene organization and homology between SpltNPV-C3 and other baculovirus genomes, additional information can be obtained to determine the diversity of baculoviruses and gene evolution. SpltNPV-C3 has 149 predicted open reading frames (ORFs), including 38 core baculovirus genes [19]. In contrast with other baculoviruses, SpltNPV-C3 shares 101 ORFs with AcMNPV, and it is estimated that these ORFs constitute approximately 67.8% of the total. Ninety-eight ORFs are homologous to SpltNPV-G2 ORFs, lower than the 103 of SeMNPV and 141 of SperNPV. SpltNPV-C3 and SpltNPV-G2 were found in the same host, but the homology between them was lower than that between SeMNPV and SperNPV. In this study, SpltNPV-C3 ORF26, ORF27, ORF28, ORF34, ORF72, ORF89, ORF90, and ORF108 were found only in SpltNPV-C3, not in SperNPV, and ORF26, ORF27, ORF34, ORF108 had no homologs in other baculoviruses (Table 1). Protein homology analysis via BLAST revealed that these four unique ORFs had no recognizable promoter. The specific functions of these proteins may be revealed in future studies.

The whole genome of SpltNPV-C3 was compared with that of SpltNPV-G2 (NC_003102), and the percentage identity was 76.42%, which was lower than that of AcMNPV (80.20%), SeMNPV (NC_002169) (84.63%) and SperNPV (NC_055502) (96.10%). These viruses belong to the *Alphabaculovirus* genus. and their names originate from their hosts. The SpltNPV-C3 genome is most closely related to the SperNPV genome. The host of SpltNPV is *Spodoptera litura*, which is distributed across Asia and Oceania. *Spodoptera eridania* is the host of SperNPV found across North America, and it will be interesting to thoroughly investigate the discrepancy between SpltNPV and SperNPV caused by regional disparity. SpltNPV is similar to other viruses. Research has shown that BmNPV (*Bombyx mori* nucleopolyhedrovirus) has 93% homology with AcMNPV but lacks homologs of Ac3, Ac7 (orf603), Ac48, Ac49, Ac70, Ac86, and Ac134. Ac7 (orf603) is related to lethal genes and cannot be found in SpltNPV-C3 either; this is a universal phenomenon in the baculovirus family likely because these viruses have the same ancestor and lost these genes during evolution. These deletions may cause differences in hosts [20].

For viruses, a stronger lethality reduces the chance of survival. Causing rapid mortality of *S. litura* larvae is a disadvantage for generating a descendant virus. Hosts can evolve defense mechanisms to protect themselves from viruses; it is thus beneficial for viral genes to mutate more quickly. Producing more mutants leads to more chances to overcome the host’s defense. There is a set of common genes that cannot be changed; these genes are called core genes, and seem to be crucial factors for some main biological functions. Core genes control the fundamental components of baculoviruses. Other genes that transform are present in different forms in different baculoviruses probably contain confer the secret of evolutionarily advantageous functions.

### Phylogenetic analysis of SpltNPV-C3

**Fig. 3:**
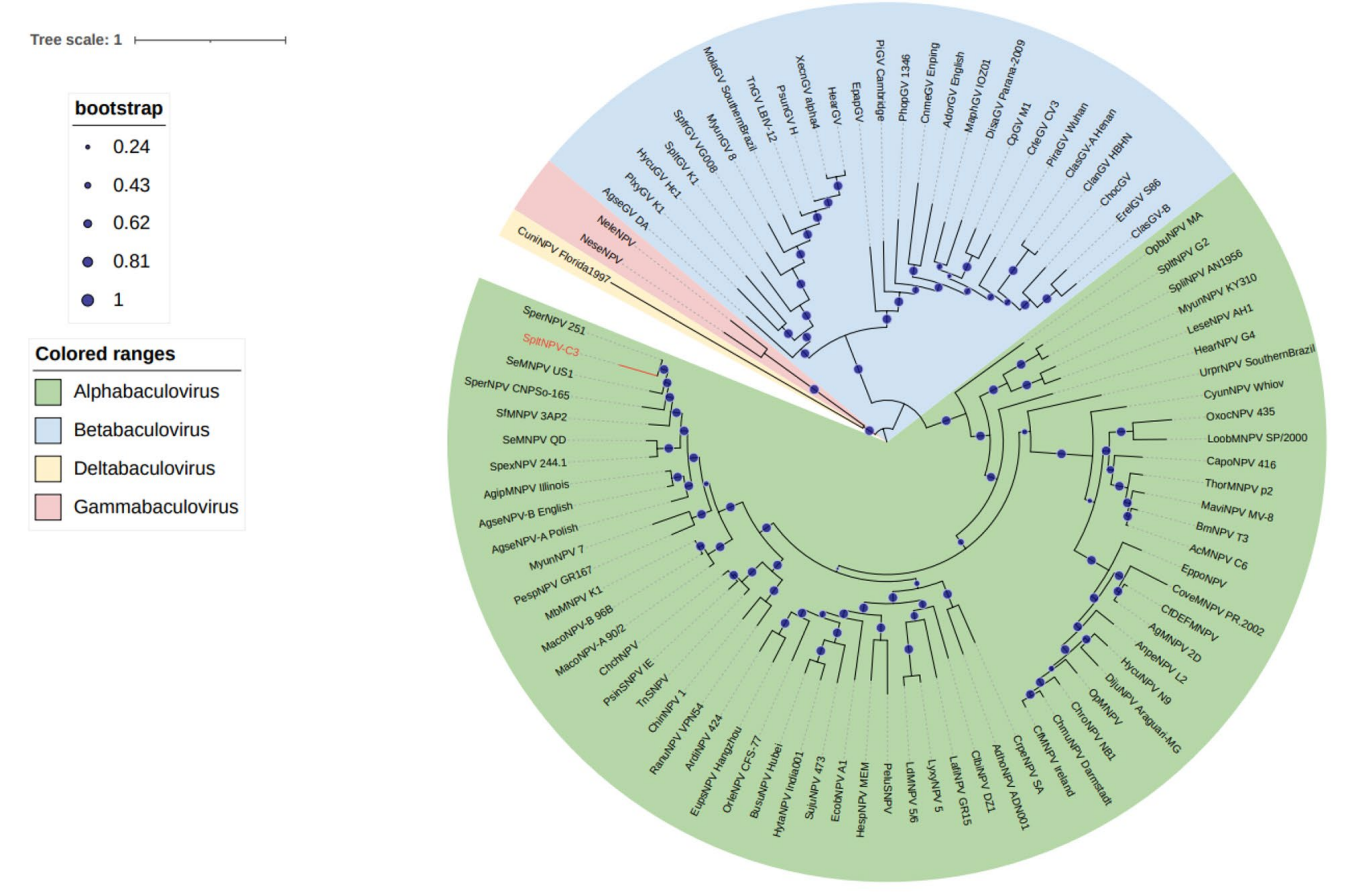
Phylogenetic tree of 90 baculoviruses with complete sequences. A phylogenetic tree was generated using MEGA X software via the maximum likelihood method and the JTT matrix-based model. The results were visualized using iTOL [21]

Genome analysis revealed 38 conserved genes in baculoviruses, all of which can be found in SpltNPV-C3. The phylogenetic analysis was based on the 38 core-gene amino acid sequences from SpltNPV-C3 and the other 89 baculoviruses that were collected and listed in ICTV (https://ictv.global/report/chapter/baculoviridae/baculoviridae) using the maximum likelihood (ML) method with 1000 bootstrap replicates. With the phylogenetic tree, SpltNPV-C3s were classified into an *Alphabaculovirus* clade, with a shorter genetic distance between SperNPV and SeMNPV. A phylogenetic tree of 90 baculoviruses with complete sequences is shown in Fig. 3.

### Virus species demarcation criteria

Traditional naming rules give precedence to the host origin. Thus, unreliable identification sometimes occurs. For example, the same virus extracted from different hosts is given a different name. With the help of molecular biology, a phylogenetic species criterion for Lepidoptera-specific baculoviruses that uses the genetic distances of the partial *lef-8*, *lef-9*, and *polh* genes has been established by an increasing number of researchers. Generally, baculoviruses are considered to belong to the same species when the distance lower than 0.015 according to the Kimura 2-parameter model [13]. The distances among AcMNPV, SeMNPV, SperNPV, SpltNPV-G2, and SpltNPV-C3 were determined. The results showed that the distance between SpltNPV-C3 and SperNPV was 0.0156, which indicated that they were closely related but still two different species. Different data were obtained when the *lef-8*, *lef-9*, and *polh* genes were separated and when calculating the genetic distance alone. The distance of was 0.0163 for *lef-8*, and 0.0222 for *lef-9*, the sequence of *polh* was identical, i.e., a distance of 0. SpltNPV-C3 and SpltNPV-G2 do not have the greatest similarity, which implies that the classifications that exist at present cannot demonstrate true relationships among baculoviruses. Interestingly, that SpltNPV-C3 and SpltNPV-G2 can infect the same insects, but their genes are not very close. In terms of the core genes, SpltNPV-C3 is very close to SperNPV, but they can infect different insects. Therefore, some important genes can influence the virus’s choice of host. Table 2 shows the distances of the nucleotide sequences.

**Table 2:** Pairwise distances of the tandemly arranged *lef8*-*lef9*-*polh* nucleotide sequences were calculated by the Kimura 2-parameter model

lef8-lef9-polh	AcMNPV	SeMNPV	SperNPV	SpltNPV-G2	SpltNPV-C3
**AcMNPV**		0.3377	0.3348	0.3473	0.3373
**SeMNPV**	0.3377		0.0656	0.3183	0.0645
**SperNPV**	0.3348	0.0656		0.3138	0.0156
**SpltNPV-G2**	0.3473	0.3183	0.3138		0.3129
**SpltNPV-C3**	0.3373	0.0645	0.0156	0.3129	

### Protein analysis via LC‒MS/MS and structure prediction

**Table 3:** Identification of the ODV proteins of SpltNPV-C3.

ORF	Protein	Characteristics/function
1(ac8)	Polyhedrin	Occlusion bodies (OBs) matrix protein
7(ac148)	odv-e56(PIF-5)	Core gene; recombinant baculovirus
11(ac130)	gp16	Da primary translation product
12(ac129)	p24	Viral capsid protein
17(ac127)	cath	Viral cathepsin-like protein
23(ac131)	ptp2	Dual-specificity phosphatase domain
36(ac22)	PIF-2	Core gene; *Per os* infectivity factor 2
37(ac119)	PIF-1	Core gene; *Per os* infectivity factor 1
47(ac131)	Calyx/pep	Polyhedral envelope protein
53(ac115)	PIF-3	Core gene; *Per os* infectivity factor 3
61(ac46)	odv-e66	Occlusion-derived virus envelope protein E66
63(ac109)	odv-ec43	Core gene; Da primary translation product
65(ac104)	p87	Viral capsid associated protein
66(ac103)	p48	Core gene; BV production and ODV envelopment
67(ac102)	p12	Hypothetical protein
68(ac101)	p40	Core gene; Subunit of protein complex
71(ac98)	38 K	Core gene; 38 kDa protein;Required for nucleocapsid assembly
77(ac94)	odv-e25	Core gene; occlusion-derived virus envelope protein
81(ac89)	vp39	Core gene; major viral capsid protein
84(ac82)	tlp20	Telokin-like protein-20
88(ac77)	vlf-1	Core gene; similarity to integrase/recombinase domain;
96(ac66)	desmop	Core gene; Baculovirus desmoplakin-like protein
110(ac54)	vp1054	Core gene; viral capsid associated protein
112	unknown	Protein of unknown function
113(se107)	unknown	Protein of unknown function
120(ac46)	odv-e66	Structural protein of ODV envelope
126(ac36)	39 K	Nuclear matrix associated phosphoprotein
129(se123)	ubiquitin	Viral ubiquitin
131(ac26)	unknown	Protein of unknown function
132(ac25)	dbp	DNA binding protein
135(ac136)	p26	Da primary translation product
136(se130)	p10	Nucleopolyhedrovirus fibrous body protein
144(ac144)	odv-e27	Core gene; occlusion-derived virus envelope/capsid protein
146(ac142)	p49	Core gene; Required for BV production

Table 3 shows the identified ODV proteins of SpltNPV-C3. According to previous research, the multiprotein complex of *per os* infectivity factors (PIFs) is indispensable for baculovirus infection of insect midgut cells. odv-e56, PIF-1, PIF-2, PIF-3, odv-ec43, p48, p40, 38 K, odv-e25, vp39, vlf-1, desmop, vp1054, odv-e27, and p49 are core genes and were detected by LC‒MS/MS, a tool for routine protein identification. Purified ODVs were separated via SDS–PAGE, and the resulting peptides were analyzed via LC‒MS/MS. Thirty-four proteins were identified, 15 of which were core baculovirus genes [22]. Interestingly, PIF-1, PIF-2, PIF-3, and PIF-4 can form a complex, but only PIF-1, PIF-2, and PIF-3 were detected in our LC‒MS/MS results, where the disposition of PIF-4 is unknown. The PIF-4 protein was not detected via LC‒MS/MS, possibly due to its low expression level.

odv-e56 (PIF-5) is also an important protein for baculoviral oral infectivity (Li, et al., 2022). This paper reveals the essential role of intramolecular interactions in baculoviral oral infectivity. 10.1128/jvi.00806 − 22). Other PIF proteins were not detected by LC‒MS/MS, and these proteins may be the cause of larval infection during the OB period.

The following ten PIF proteins from baculovirus have been authenticated: PIF-1 (ac119), PIF-2 (ac22), PIF-3 (ac115), PIF-4 (ac96), PIF-5 (odv-e56/ac148), PIF-6 (ac68), PIF-7 (ac110), P95 or PIF-8 (ac83) [23, 24] and PIF-9 [24]. PIF-1, PIF-2, PIF-3, and PIF-4 can form a stable complex. PIF-4 is essential for oral infectivity in AcMNPV, but it is not stable in the PIF- complex. When PIF-4 is deleted, PIF-1, PIF-2, and PIF-3 can form a smaller complex. PIF-4 was not detected in our LC‒MS/MS analysis, and may be separately involved in the process of infection. PIF-1 and PIF-2 seemed to mediate ODV-binding in a species specific manner, when AcMNPV or SpltNPV-C3 PIF-1, PIF-2, and PIF-3 were used in place of PIFs in HearNPV (*Helicoverpa armigera* nucleopolyhedrovirus), these viruses lost oral infectivity, with the exception of SpltNPV-C3 PIF-3 [25].

**Fig. 4:**
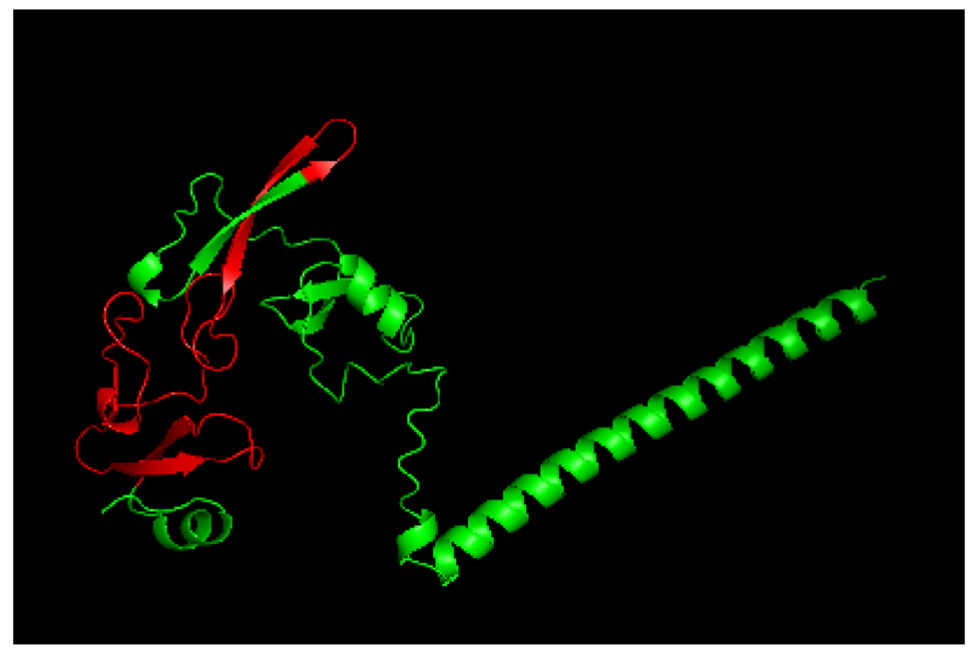
Conserved sequence in SpltNPV-C3 PIF-3. The 3D structure was predicted by AlphaFold 2, and the conserved sequence was calculated by ConSurf.

This result is interesting because it shows that PIF-1 and PIF-2 are related to recognizing the host and that some parts of PIF-3 can help the virus infect midgut cells. We simulated the 3D structure of SpltNPV-C3 PIF-1, PIF-2, PIF-3, and PIF-4 and calculated the conserved amino acids by multiple sequence alignment on The ConSurf Server (tau.ac.il). After contrasting AcMNPV, BmNPV, SperNPV, SeMNPV, and SpltNPV-G2, we discovered a conserved sequence on the tail of PIF-3. The structure predicted by AlphaFold 2 is shown in Fig. 4.

**Fig. 5:**
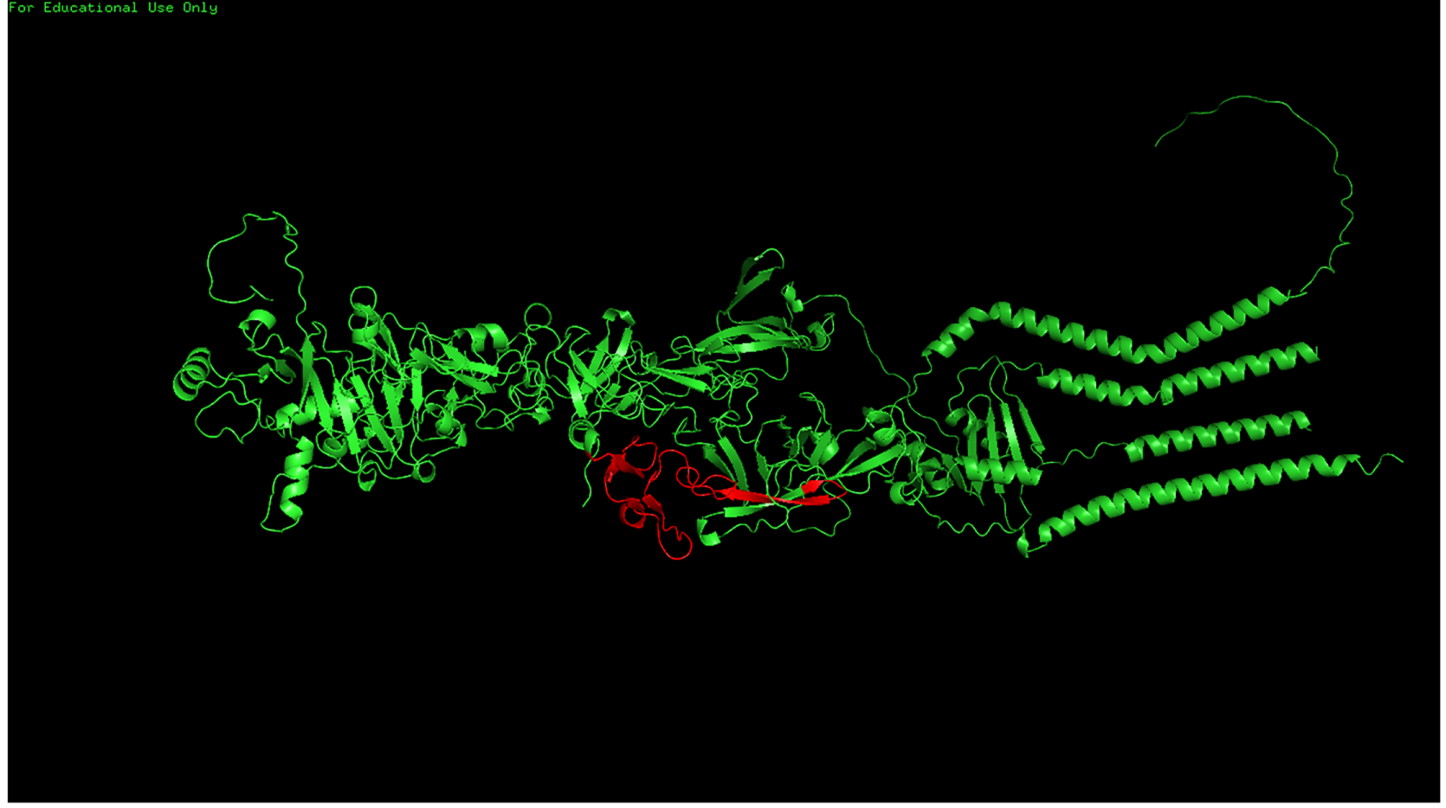
3D structures of the PIF-1, PIF-2, PIF-3, and PIF-4 complex simulated by the AlphaFold multimer tool

After the PIF-3 model was constructed, the AlphaFold Multimer tool was used to predict the model of the PIF-1, PIF-2, PIF-3, and PIF-4 complex. The red region is a conserved sequence located in the middle of the PIF complex. These amino acids are preserved throughout evolution, and thus this region may correlate well with the infecting larval midgut. The structure of the PIF-1, PIF-2, PIF-3, and PIF-4 complexes were simulated by the AlphaFold Multimer tool, as shown in Fig. 5.

## Conclusion

The morphological characteristics of purified OBs and ODVs of SpltNPV-C3 were examined morphological characteristics under EM. The OBs of SpltNPV-C3 are approximately 1.5 μm in diameter, and the ODV is approximately 300 nm in length and 40 nm in width. The whole genome of SpltNPV-C3 is 148,634 bp (GenBank accession 780,426), with a G + C content of 45%, and 149 ORFs were found. Using the ML method, a phylogenetic tree of 90 baculoviruses was constructed, and SpltNPV-C3 was found to belong to the *Alphabaculovirus* group and was most closely related to SperNPV according to our tree. Thirty-four proteins were found in the purified ODVs, 15 of which were core genes in the family *Baculoviridae*. The complex of PIF-1, PIF-2, PIF-3, and PIF-4 was simulated by the AlphaFold Multimer tool, and a conserved sequence of PIF-3 was found in the middle of the PIF complex. This research is helpful for studying baculovirus infection and the origin of the baculovirus family.

### Electronic supplementary material

Below is the link to the electronic supplementary material.

Supplementary Material 1

## Data Availability

The datasets generated and/or analysed during the current study are not publicly available due [REASON WHY DATA ARE NOT PUBLIC] but are available from the corresponding author on reasonable request.

## Abbreviations

SpltNPV: *Spodoptera litura* nucleopolyhedrovirus
OBs: occlusion bodies
ODVs: occlusion-derived viruses
SEM: scanning electron microscopy
TEM: transmission electron microscopy
PIFs: *per os* infectivity factors
AcMNPV: *Autographa California* multiple nucleopolyhedroviruses
AgseNPV: *Agrotis segetum* nucleopolyhedrovirus
SeMNPV: *Spodoptera exigua* multiple nucleopolyhedrovirus
SperNPV: *Spodoptera eridania* nucleopolyhedrovirus

## References

[1] Harrison RL, Herniou EA, Jehle JA, Theilmann DA, Burand JP, Becnel JJ, Krell PJ, van Oers MM, Mowery JD (2018). Bauchan GR and Ictv Report C: ICTV Virus Taxonomy Profile: Baculoviridae. J Gen Virol.

[2] Hofmann C, Sandig V, Jennings G, Rudolph M, Schlag P, Strauss M (1995). Efficient gene-transfer into human hepatocytes by Baculovirus vectors. P Natl Acad Sci USA.

[3] Jehle JA, Blissard GW, Bonning BC, Cory JS, Herniou EA, Rohrmann GF, Theilmann DA, Thiem SM, Vlak JM (2006). On the classification and nomenclature of baculoviruses: a proposal for revision. Arch Virol.

[4] Wang XF, Zhang BQ, Xu HJ, Cui YJ, Xu YP, Zhang MJ, Han YS, Lee YS, Bao YY, Zhang CX (2011). ODV-associated proteins of the Pieris rapae Granulovirus. J Proteome Res.

[5] Zwart MP, Ali G, Strien EAV, Schijlen E, Wang M, Werf WV, Vlak JM. Identification of Loci Associated with Enhanced Virulence in Spodoptera litura Nucleopolyhedrovirus Isolates Using Deep Sequencing. *Viruses* 2019, 11. 10.3390/v11090872 PMC6783950 31533344

[6] Kamiya K, Zhu J, Murata M, Laviña-Caoili BA, Ikeda M, Kobayashi M, Kawamura S (2004). Cloning and comparative characterization of three distinct nucleopolyhedroviruses isolated from the common cutworm, Spodoptera litura (Lepidoptera: Noctuidae) in Japan. Biol Control.

[7] Xu F, Ince IA, Boeren S, Vlak JM, van Oers MM (2011). Protein composition of the occlusion derived virus of Chrysodeixis chalcites nucleopolyhedrovirus. Virus Res.

[8] Tang P, Zhang H, Li YN, Han B, Wang GZ, Qin QL, Zhang ZF. Genomic sequencing and analyses of HearMNPV-a new Multinucleocapsid Nucleopolyhedrovirus isolated from Helicoverpa armigera. Virol J 2012, 9. 10.1186/1743-422X-9-168 PMC3545888 22913743

[9] Chen Y, Lin X, Yi YZ, Lu YY, Zhang ZF (2009). Construction and application of a Baculovirus genomic Library. Z Naturforsch C.

[10] Zimmermann L, Stephens A, Nam SZ, Rau D, Kubler J, Lozajic M, Gabler F, Soding J (2018). Lupas AN and Alva V: a completely reimplemented MPI Bioinformatics Toolkit with a new HHpred server at its core. J Mol Biol.

[11] Soding J, Biegert A, Lupas AN (2005). The HHpred interactive server for protein homology detection and structure prediction. Nucleic Acids Res.

[12] Kool M, Vlak JM (1993). The structural and functional organization of the Autographa californica nuclear polyhedrosis virus genome. Arch Virol.

[13] Jehle JA, Lange M, Wang H, Hu Z, Wang Y, Hauschild R (2006). Molecular identification and phylogenetic analysis of baculoviruses from Lepidoptera. Virology.

[14] Liu XJ, Li YN, Hu XY, Yi YZ, Zhang ZF (2017). Gene delivery and gene expression in vertebrate using baculovirus Bombyx mori nucleopolyhedrovirus vector. Oncotarget.

[15] Hofmann C, Strauss M (1998). Baculovirus-mediated gene transfer in the presence of human serum or blood facilitated by inhibition of the complement system. Gene Ther.

[16] Pang Y, Yu J, Wang L, Hu X, Bao W, Li G, Chen C, Han H, Hu S, Yang H (2001). Sequence analysis of the Spodoptera litura multicapsid nucleopolyhedrovirus genome. Virology.

[17] Hashimoto Y, Hayakawa T, Ueno Y, Fujita T, Sano Y, Matsumoto T (2000). Sequence analysis of the Plutella xylostella granulovirus genome. Virology.

[18] WF IJ, Westenberg M, Goldbach RW, Blissard GW, Vlak JM, Zuidema D (2000). A novel baculovirus envelope fusion protein with a proprotein convertase cleavage site. Virology.

[19] Wang M, Hu Z (2020). Advances in Molecular Biology of Baculoviruses. Curr Issues Mol Biol.

[20] Gomi S, Majima K, Maeda S (1999). Sequence analysis of the genome of Bombyx mori nucleopolyhedrovirus. J Gen Virol.

[21] Li Y, Liu X, Tang P, Zhang H, Qin Q, Zhang Z (2021). Genome sequence and organization of the Mythimna (formerly Pseudaletia) unipuncta granulovirus hawaiian strain. Sci Rep.

[22] Garavaglia MJ, Miele SA, Iserte JA, Belaich MN, Ghiringhelli PD (2012). The ac53, ac78, ac101, and ac103 genes are newly discovered core genes in the family Baculoviridae. J Virol.

[23] Boogaard B, van Lent JWM, Theilmann DA, Erlandson MA, van Oers MM (2017). Baculoviruses require an intact ODV entry-complex to resist proteolytic degradation of per os infectivity factors by co-occluded proteases from the larval host. J Gen Virol.

[24] Wang X, Shang Y, Chen C, Liu S, Chang M, Zhang N, Hu H, Zhang F, Zhang T, Wang Z, Liu X, Lin Z, Deng F, Wang H, Zou Z, Vlak JM, Wang M, Hu Z. Baculovirus Per Os infectivity factor complex: components and Assembly. J Virol 2019, 93. 10.1128/JVI.02053-18 PMC6401453 30602603

[25] Boogaard B, van Oers MM, van Lent JWM. An Advanced View on Baculovirus per Os Infectivity Factors. *Insects* 2018, 9. 10.3390/insects9030084 PMC6164829 30018247

