# Supplementary material for: Genome characteristics and the ODV proteome of a second distinct alphabaculovirus from Spodoptera litura
Source: BMC Genomics. 2024 Jan 22;25:91. doi: 10.1186/s12864-024-09989-3 (PMC10804782; doi:10.1186/s12864-024-09989-3)
Supplement: Supplementary file 1 — Supplementary Material 1 [file 12864_2024_9989_MOESM1_ESM.docx]

Table S1. Basic information of 93 sequenced baculovirus genomes in GenBank

| Genus | Genome | Abbreviation | Accession | Source information | Length | Protein | Neighbors | *hrs* | *bros* |
| --- | --- | --- | --- | --- | --- | --- | --- | --- | --- |
| Alphabaculovirus (63) | Adoxophyes honmai nucleopolyhedrovirus | AdhoNPV | NC_004690 | strain:ADN001 | 113220 | 125 | - | 4 | 4 |
|  | Adoxophyes orana nucleopolyhedrovirus | AdorNPV | NC_011423 | isolate:English | 111724 | 121 | - | 4 | 3 |
|  | Agrotis ipsilon multiple nucleopolyhedrovirus | AgipMNPV | NC_011345 | strain:Illinois | 155122 | 163 | - | 7 | 5 |
|  | Agrotis segetum nucleopolyhedrovirus A | AgseNPV-A | NC_007921 |  | 147544 | 153 | - | 5 | 4 |
|  | Agrotis segetum nucleopolyhedrovirus B | AgseNPV-B | NC_025960 | isolate:English | 148981 | 150 | - | 6 | 2 |
|  | Antheraea pernyi nucleopolyhedrovirus | AnpeNPV | NC_008035 | strain:Liaoning | 126629 | 147 | 7 | 3 | 2 |
|  | Anticarsia gemmatalis multiple nucleopolyhedrovirus | AngeMNPV | NC_031761 | isolate:AgMNPV-37 | 131855 | 156 | 2 | 11 | 6 |
|  | Anticarsia gemmatalis nucleopolyhedrovirus | AngeNPV | NC_008520 | isolate:AgMNPV-2D | 132239 | 158 | - | 9 | 7 |
|  | Apocheima cinerarium nucleopolyhedrovirus | ApciNPV | NC_018504 |  | 123876 | 117 | - | 4 | 1 |
|  | Autographa californica nucleopolyhedrovirus | AcMNPV | NC_001623 |  | 133894 | 156 | 6 | 9 | 1 |
|  | Rachiplusia ou MNPV |  | NC_004323 |  | 131526 | 149 | - | 9 | 0 |
|  | Bombyx mori nucleopolyhedrovirus | BmNPV | NC_001962 | isolate:T3 | 128413 | 143 | 11 | 7 | 5 |
|  | Buzura suppressaria nucleopolyhedrovirus | BusuNPV | NC_023442 | isolate:Hubei | 120420 | 127 | 1 | 0 | 3 |
|  | Catopsilia pomona nucleopolyhedrovirus | CapoNPV | NC_030240 | isolate:416 | 128058 | 130 | - | 8 | 1 |
|  | Choristoneura fumiferana DEF multiple nucleopolyhedrovirus | CfDEFMNPV | NC_005137 |  | 131160 | 149 | - | 13 | 4 |
|  | Choristoneura fumiferana multiple nucleopolyhedrovirus | CfMNPV | NC_004778 |  | 129593 | 146 | 1 | 5 | 1 |
|  | Choristoneura occidentalis alphabaculovirus |  | NC_021925 | isolate:BC_1 | 128446 | 148 | - | 5 | 2 |
|  | Choristoneura murinana nucleopolyhedrovirus | ChmuNPV | NC_023177 | strain:Darmstadt | 124688 | 147 | - | 2 | 1 |
|  | Choristoneura rosaceana alphabaculovirus | ChroNPV | NC_021924 | isolate:NB_1 | 129052 | 149 | - | 3 | 2 |
|  | Chrysodeixis chalcites nucleopolyhedrovirus | ChchNPV | NC_007151 |  | 149622 | 151 | 5 | 0 | 4 |
|  | Clanis bilineata nucleopolyhedrovirus | ClbiNPV | NC_008293 | isolate:DZ1 | 135454 | 129 | - | 0 | 3 |
|  | Condylorrhiza vestigialis MNPV | CoveMNPV | NC_026430 |  | 125767 | 138 | - | 4 | 9 |
|  | Cryptophlebia peltastica nucleopolyhedrovirus |  | NC_055500 | isolate:SA | 115728 | 126 | - | 5 | 3 |
|  | Cyclophragma undans nucleopolyhedrovirus |  | NC_055467 | isolate:Whiov | 142900 | 147 | - | 14 | 10 |
|  | Ectropis obliqua nucleopolyhedrovirus | EcobNPV | NC_008586 | strain:A1 | 131204 | 126 | 1 | 3 | 2 |
|  | Epiphyas postvittana nucleopolyhedrovirus | EppoNPV | NC_003083 |  | 118584 | 136 | - | 5 | 1 |
|  | Euproctis pseudoconspersa nucleopolyhedrovirus | EupsNPV | NC_012639 | strain:Hangzhou | 141291 | 139 | - | 4 | 2 |
|  | Helicoverpa armigera multiple nucleopolyhedrovirus |  | NC_011615 |  | 154196 | 162 | - | 4 | 6 |
|  | Mamestra configurata nucleopolyhedrovirus B | MacoNPV-B | NC_004117 |  | 158482 | 168 | 2 | 4 | 7 |
|  | Helicoverpa armigera nucleopolyhedrovirus | HearNPV | NC_003094 | isolate:C1 | 130759 | 137 | 18 | 5 | 3 |
|  | Helicoverpa armigera nucleopolyhedrovirus G4 | HearNPV-G4 | NC_002654 |  | 131405 | 135 | 18 | 5 | 3 |
|  | Hemileuca sp. Nucleopolyhedrovirus | HespNPV | NC_021923 |  | 140633 | 137 | - | 3 | 2 |
|  | Hyphantria cunea nucleopolyhedrovirus | HycuNPV | NC_007767 |  | 132959 | 148 | - | 6 | 5 |
|  | Hyposidra talaca NPV |  | NC_055453 | strain:HytaNPVIndia001 | 139089 | 141 | - | 6 | 4 |
|  | Lambdina fiscellaria nucleopolyhedrovirus | LafiNPV | NC_026922 | isolate:GR15 | 157977 | 137 | - | 2 | 0 |
|  | Leucania separata nucleopolyhedrovirus | LeseNPV | NC_008348 | strain:AH1 | 168041 | 169 | - | 8 | 10 |
|  | Lonomia obliqua multiple nucleopolyhedrovirus | LoobNPV | NC_043520 |  | 120023 | 134 | - | 7 | 1 |
|  | Lymantria dispar multiple nucleopolyhedrovirus | LdMNPV | NC_001973 |  | 161046 | 164 | 13 | 13 | 16 |
|  | Lymantria xylina nucleopolyhedrovirus | LyxyMNPV | NC_013953 | isolate:LyxyMNPV-5 | 156344 | 157 | - | 13 | 14 |
|  | Malacosoma neustria nucleopolyhedrovirus | ManeNPV | NC_040606 | isolate:ManeNPV-T2 | 130202 | 131 | - | 0 | 2 |
|  | Mamestra brassicae multiple nucleopolyhedrovirus | MabrMNPV | NC_023681 | strain:K1 | 152710 | 159 | 2 | 4 | 6 |
|  | Mamestra configurata nucleopolyhedrovirus A | MacoNPV-A | NC_003529 | strain:90/2 | 155060 | 169 | 1 | 4 | 8 |
|  | Maruca vitrata nucleopolyhedrovirus | MaviNPV | NC_008725 |  | 111953 | 126 | - | 5 | 0 |
|  | Mythimna unipuncta nucleopolyhedrovirus | MyunNPV | NC_043530 |  | 148482 | 158 | - | 6 | 7 |
|  | Operophtera brumata nucleopolyhedrovirus | OpbuNPV | NC_040621 | isolate:OpbuNPV-MA | 119054 | 130 | - | 5 | 2 |
|  | Orgyia leucostigma nucleopolyhedrovirus | OrleNPV | NC_010276 | isolate:CFS-77 | 156179 | 135 | - | 3 | 5 |
|  | Orgyia pseudotsugata multiple nucleopolyhedrovirus | OpMNPV | NC_001875 |  | 131995 | 152 | - | 5 | 3 |
|  | Oxyplax ochracea nucleopolyhedrovirus | OxocNPV | NC_043529 |  | 113971 | 124 | - | 6 | 0 |
|  | Peridroma alphabaculovirus | PespNPV | NC_024625 | isolate:GR_167 | 151109 | 139 | - | 2 | 6 |
|  | Perigonia lusca single nucleopolyhedrovirus | PeluSNPV | NC_027923 |  | 132831 | 145 | - | 2 | 1 |
|  | Pseudoplusia includens SNPV IE | PsinSNPV | NC_026268 |  | 139132 | 141 | 6 | 0 | 2 |
|  | Spodoptera eridania nucleopolyhedrovirus | SperNPV | NC_055502 | isolate:251 | 149090 | 146 | 1 | 0 | 2 |
|  | Spodoptera exempta nucleopolyhedrovirus | SeNPV | NC_055455 | strain:244.1 | 129528 | 139 | - | 5 | 2 |
|  | Spodoptera exigua multiple nucleopolyhedrovirus | SeMNPV | NC_002169 |  | 135611 | 139 | 8 | 6 | 0 |
|  | Spodoptera frugiperda multiple nucleopolyhedrovirus | SfMNPV | NC_009011 | isolate:3AP2 | 131331 | 143 | 4 | 8 | 1 |
|  | Spodoptera littoralis nucleopolyhedrovirus | SpliMNPV | NC_038369 | isolate:AN1956 | 137998 | 132 | 1 | 15 | 1 |
|  | Spodoptera litura nucleopolyhedrovirus | SpltNPV | NC_003102 | strain:G2 | 139342 | 141 | - | 17 | 2 |
|  | Spodoptera litura nucleopolyhedrovirus II | SpltNPV-II | NC_011616 |  | 148634 | 147 | - | 7 | 2 |
|  | Sucra jujuba nucleopolyhedrovirus | SujuNPV | NC_028636 | isolate:473 | 135952 | 131 | - | 7 | 4 |
|  | Thysanoplusia orichalcea nucleopolyhedrovirus | ThorNPV | NC_019945 | isolate:p2 | 132978 | 145 | 1 | 6 | 2 |
|  | Trichoplusia ni single nucleopolyhedrovirus | TnSNPV | NC_007383 |  | 134394 | 145 | - | 0 | 2 |
|  | Urbanus proteus nucleopolyhedrovirus | UrprNPV | NC_029997 | isolate:Southern Brazil | 105555 | 119 | - | 0 | 7 |
|  | Wiseana signata nucleopolyhedrovirus **(incomplete)** | WisiSNPV | NC_038370 | strain:WisiSNPV | 1182 | 1 | - | - | - |
| Betabaculovirus (26) | Adoxophyes orana granulovirus | AdorGV | NC_005038 |  | 99657 | 119 | 1 | 0 | 0 |
|  | Agrotis segetum granulovirus | AgseGV | NC_039213 | strain:DA | 131557 | 152 | 2 | 2 | 1 |
|  | Artogeia rapae granulovirus | ArGV | NC_013797 | isolate:Wuhan | 108592 | 120 | - | 8 | 0 |
|  | Choristoneura fumiferana granulovirus | ChfuGV | NC_008168 |  | 104710 | 116 | - | 5 | 0 |
|  | Clostera anachoreta granulovirus | ClanGV | NC_015398 | isolate:ClanGV-HBHN | 101487 | 123 | - | 4 | 0 |
|  | Clostera anastomosis granulovirus B | ClasGV-B | NC_038371 | ClasGV-B | 107439 | 123 | - | 0 | 0 |
|  | Clostera anastomosis granulovirus Henan | ClasGV-Henan | NC_022646 | isolate:CaLGV-Henan | 101818 | 122 | - | 1 | 0 |
|  | Cnaphalocrocis medinalis granulovirus | CnmeGV | NC_029304 | strain:Enping | 111246 | 118 | 1 | 0 | 3 |
|  | Cryptophlebia leucotreta granulovirus | CrleGV | NC_005068 | isolate:CV3 | 110907 | 128 | 1 | 3 | 0 |
|  | Cydia pomonella granulovirus | CpGV | NC_002816 | strain:Mexican 1 | 123500 | 143 | 5 | 0 | 1 |
|  | Diatraea saccharalis granulovirus | DisaGV | NC_028491 | strain:Parana-2009; isolate:DisaGV-Parana-2009 | 98392 | 125 | - | 7 | 0 |
|  | Epinotia aporema granulovirus | EpapGV | NC_018875 |  | 119082 | 132 | - | 16 | 0 |
|  | Erinnyis ello granulovirus | ErelGV | NC_025257 | isolate:S86 | 102759 | 130 | 6 | 5 | 0 |
|  | Harrisina brillians granulovirus **(incomplete)** | HbGV | NC_038372 |  | 1241 | 3 |  | - | - |
|  | Helicoverpa armigera granulovirus | HearGV | NC_010240 |  | 169794 | 179 | - | 9 | 10 |
|  | Lacanobia oleracea granulovirus **(incomplete)** | LoGV | NC_038868 | substrain:LOGV-S1; isolate:Scottish | 2108 | 1 |  | - | - |
|  | Mocis latipes granulovirus | MolaGV | NC_029996 | isolate:Southern Brazil | 134272 | 145 | - | 2 | 2 |
|  | Mythimna (Pseudaletia) unipuncta granulovirus | MyunGV-A | NC_013772 | strain:Hawaiin | 176677 | 183 | - | 9 | 12 |
|  | Mythimna unipuncta granulovirus B | MyunGV-B | NC_033780 | isolate:MyunGV#8 | 144673 | 153 | - | 6 | 5 |
|  | Phthorimaea operculella granulovirus | PhopGV | NC_004062 |  | 119217 | 130 | 1 | 12 | 1 |
|  | Plodia interpunctella granulovirus | PlinGV | NC_032255 | isolate:Cambridge | 112536 | 123 | - | 7 | 0 |
|  | Plutella xylostella granulovirus | PlxyGV | NC_002593 | strain:K1 | 100999 | 120 | 5 | 4 | 0 |
|  | Spodoptera frugiperda granulovirus | SpfrGV | NC_026511 | isolate:VG008 | 140913 | 146 | 1 | 8 | 7 |
|  | Spodoptera litura granulovirus | SpltGV | NC_009503 | isolate:SlGV-K1 | 124121 | 136 | - | 0 | 6 |
|  | Trichoplusia ni granulovirus LBIV-12 | TnGV | NC_038375 | isolate:LBIV-12 | 175360 | 172 | - | - | 11 |
|  | Xestia c-nigrum granulovirus | XcGV | NC_002331 |  | 178733 | 181 | - | 9 | 7 |
| Deltabaculovirus | Culex nigripalpus nucleopolyhedrovirus | CuniNPV | NC_003084 | isolate:Florida1997 | 108252 | 109 | - | 4 | 6 |
| Gammabaculovirus | Neodiprion abietis NPV | NeabNPV | NC_008252 |  | 84264 | 93 | - | 5 | 0 |
|  | Neodiprion lecontei nucleopolyhedrovirus | NeleNPV | NC_005906 |  | 81755 | 89 | - | 0 | 0 |
|  | Neodiprion sertifer nucleopolyhedrovirus | NeseNPV | NC_005905 |  | 86462 | 90 | - | 6 | 0 |

Baculoviridae - 93 genomes (include 3 incomplete genomes)

Alphabaculovirus [63] (1 incomplete); Betabaculovirus [26] (2 incomplete); Deltabaculovirus [1]; Gammabaculovirus [3]; unclassified Baculoviridae [1]

Updated by Dec 26, 2021. https://www.ncbi.nlm.nih.gov/genomes/GenomesGroup.cgi?opt=virus&taxid=10442
